# Christchurch shallow groundwater quality survey dataset

**DOI:** 10.1016/j.dib.2022.107982

**Published:** 2022-02-21

**Authors:** Irene Setiawan, Leanne Morgan, Crile Doscher, Kelvin Ng, Amandine Bosserelle

**Affiliations:** a Department of Environment, Society and Design, Lincoln University, Lincoln 7647, New Zealand; b Waterways Centre for Freshwater Management, University of Canterbury, Private Bag 4800, Christchurch, New Zealand; c College of Science and Engineering, Flinders University, GPO Box 2100, Adelaide SA 5001, Australia; d Tonkin & Taylor Limited, 51 Halifax Street, Nelson 7010, New Zealand; e Department of Civil and Natural Resources Engineering, University of Canterbury, Ilam, Christchurch, New Zealand; f WSP, 12 Moorhouse Avenue, Addington, Christchurch 8011, New Zealand

**Keywords:** Water table, Specific conductance, Temperature, pH, Chloride, Alkalinity, Dissolved oxygen, Water level

## Abstract

Shallow groundwater quality and level across the low-lying coastal city of Christchurch, New Zealand were surveyed at a high spatial resolution (1.3 piezometers/km^2^) in the spring of 2020. The groundwater quality parameters recorded across 99 piezometers include specific conductance, temperature, pH, and dissolved oxygen, following the pumping of approximately three bore volumes. Additionally, 27 out of 99 piezometers were analysed for chloride concentration and alkalinity as calcium carbonate. This dataset is useful to explore shallow groundwater conditions and how these might impact co-existing subsurface infrastructure and ecosystems. Furthermore, this dataset provides a valuable point of comparison against future changes, for example due to increased seawater intrusion, pollution events, or groundwater level rise.

## Specifications Table

**Table:** 

Subject	Hydrology and Water quality
Specific subject area	Coastal Hydrogeology
Type of data	Table
How the data were acquired	Shallow groundwater quality parameters (specific conductance at a reference temperature of 25°C, temperature, pH, and dissolved oxygen) were recorded following the pumping of approximately three bore volumes from 99 piezometers in Christchurch, New Zealand in the spring of 2020. Out of the 99 piezometers measured, 27 piezometers were sampled for chloride concentration and alkalinity as calcium carbonate.
Data format	Raw Analyzed
Description of data collection	All sampled piezometers are screened within the Christchurch Formation unconfined coastal aquifer. The sampling density was based on the proximity to tidal water features. The inland boundary of the sampled area was based on the five-meter thickness contour line of the Christchurch Formation from [1].
Data source location	• *City/Town/Region:* Christchurch • *Country:* New Zealand • *Latitude and longitude (and GPS coordinates, if possible) for collected samples/data:* Shown within the dataset • *Secondary data:* The latitude and longitude of piezometers [2] and tide predictions [3]
Data accessibility	Repository name: Mendeley Data Data identification number: https://www.doi.org/10.17632/yzmxssvn69.1 Direct URL to data: https://www.doi.org/10.17632/yzmxssvn69.1
Related research article	Setiawan, I., Morgan, L., Doscher, C., Ng, K., & Bosserelle, A. (2022). Mapping shallow groundwater salinity in a coastal urban setting to assess exposure of municipal assets. *Journal of Hydrology: Regional Studies, 40*, 100999. https://doi.org/10.1016/j.ejrh.2022.100999

## Value of the Data

• This dataset is useful to provide information on shallow groundwater quality in the low-lying coastal city of Christchurch, New Zealand, where no city-wide shallow groundwater quality survey has been undertaken previously. This was made possible by the uniquely extensive piezometer network in Christchurch installed following the Canterbury Earthquake Sequence in 2010-11 to assess liquefaction risk.
• This dataset can be used to explore shallow groundwater conditions, and how these might impact co-existing infrastructure. Extensive underground infrastructure, e.g., subsurface pipes, road foundations, and building basements co-exist with shallow groundwater, which could cause premature deterioration depending on the aggressiveness of the groundwater environment [4].
• Groundwater-dependent ecosystems have varying tolerance to different groundwater chemistry parameters [5,6], which can be investigated using this dataset.
• Asset Managers can use this dataset to highlight potentially vulnerable infrastructure; Landscape Architects and Ecologists can use this dataset to decide what vegetation species to plant at a given location depending on their chemical tolerance thresholds and the potential exposure to shallow groundwater; Civil Engineers can use this dataset to help determine what material should be used based on its tolerance to certain groundwater conditions.
• This dataset can be used as a point of comparison for future shallow groundwater surveys in Christchurch, New Zealand, for example to investigate changes in groundwater salinity and level due to sea-level rise, increased seawater intrusion or pollution events.

## 1 Data Description

Dataset of the Christchurch shallow groundwater survey of 99 piezometers conducted from 8 September to 21 October 2020, which includes sampling date, well information (identifiers, latitude and longitude [2], casing material, measuring point description and location relative to ground level, diameter), groundwater level relative to measuring point pre- and post-pumping, high and low tide time on the day of sampling, tide condition during sampling [3], measured well depth, time of pumping commencement, sample appearance and odour at the start and end of pumping, pumping duration, proportion of 3 bore volumes pumped (approximated from a maximum pumping rate of 1 litre per 16 seconds), sample temperature post-pumping, groundwater quality parameters post-pumping (temperature, specific conductance at a reference temperature of 25°C, pH, dissolved oxygen), chloride concentration and alkalinity as calcium carbonate of 27 randomly-selected wells (“spot checks” for seawater intrusion status), and additional comments. The full secondary dataset of well information [2] and tide predictions [3] are included in the data repository.

## 2 Experimental Design, Materials and Methods

**Fig. 1:**
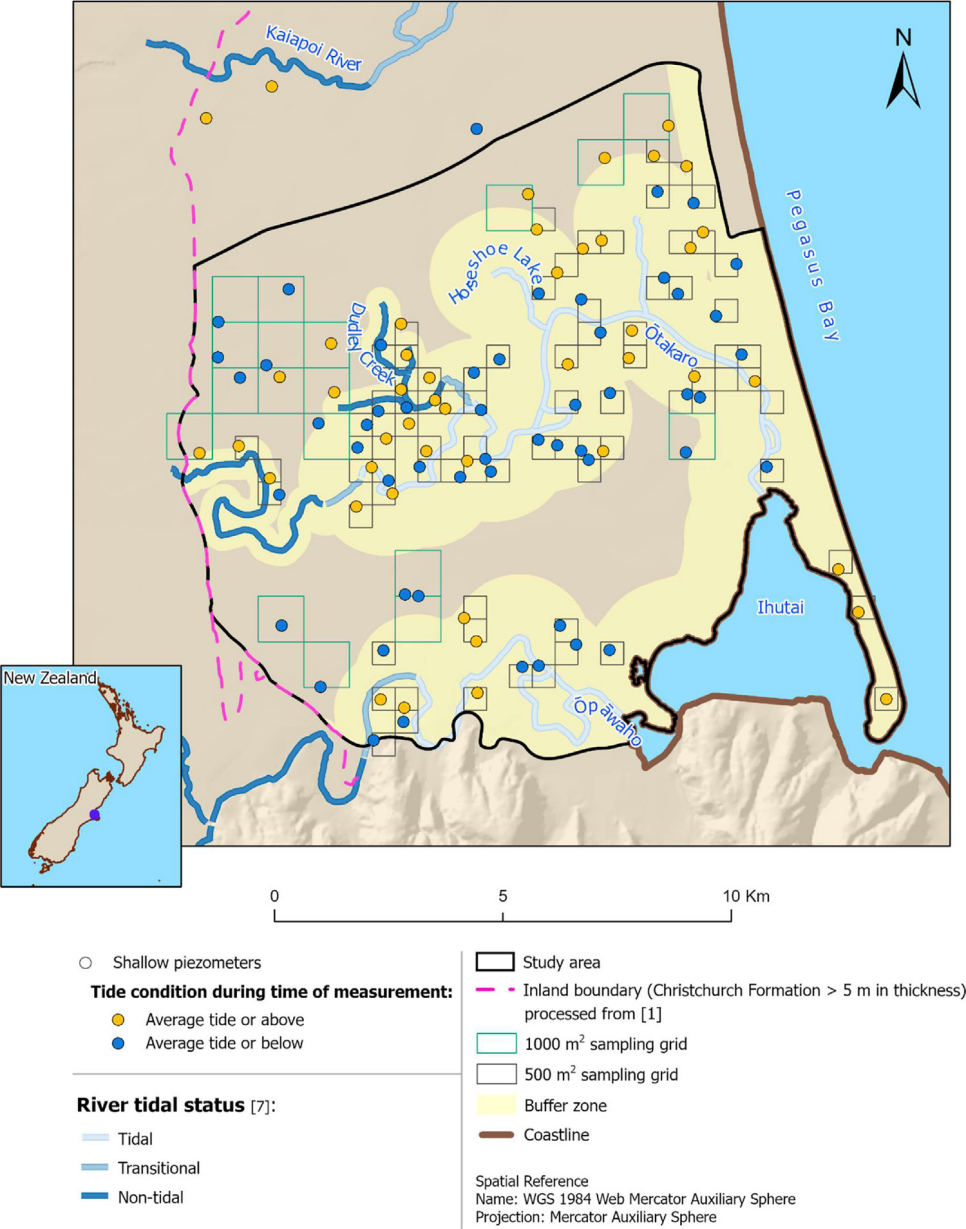
The study area within Christchurch, selected shallow piezometers (where measurements were taken in certain tide conditions) based on the 500 m^2^ sampling grid within the buffer zone and 1000 m^2^ sampling grid outside the buffer zone, river tidal status [7], inland boundary (processed from [1]), and the coastline [8]. This figure is modified from [9].

Tidal surface water bodies are a source of salinity; therefore, a higher sampling density was targeted around them. To achieve this, buffer zones were drawn around surface water bodies, which widths depend on whether the surface water body is tidal (1,000 m buffer zone), transitional (500 m buffer zone) or non-tidal (250 m buffer zone), based on the tidal categorization of [7] (Fig. 1). A sampling density of 1 piezometer per 0.5 km^2^ was applied within the buffer zone, while a sparser sampling density of 1 piezometer per 1 km^2^ was applied outside of the buffer zone. In addition, the inland boundary of the sampled area is based on the five-meter thickness contour line, processed from [1].

The sampled piezometers were drilled using the sonic method and are made of slotted PVC standpipes capped at the bottom, with depths of up to 7.5 meters below ground and screen lengths varying from 1 to 3 meters from the bottom of the piezometers [7]. One exception to this was M35/3740, which was a driven pipe made of steel [10], with a measured depth of 2.1 meters below ground.

Shallow groundwater quality parameters (specific conductance at a reference temperature of 25°C, temperature, pH, and dissolved oxygen) were recorded using YSI Professional Plus multiparameter instrument [11], connected to a YSI 6850 flow cell [12], following the pumping of approximately three bore volumes using an an Isco PTP-150 portable pump [13] from 99 piezometers in Christchurch, New Zealand in the spring of 2020. Out of the 99 piezometers sampled, unfiltered samples from 27 piezometers were analysed for alkalinity as calcium carbonate in the field using a Hach digital titrator model 16900 [14], converted into bicarbonate [15]. Filtered samples from these 27 piezometers using a 0.45 µm filter were also analysed for chloride concentration in the laboratory using a Thermo Fisher Scientific Dionex Ion Chromatograph [16]. The tide conditions during the time of sampling were described as “average tide or above” or “average tide or below”, based on tide data at Sumner beach, Christchurch, New Zealand [3].

## Ethics Statements

Our work did not involve human subjects or animal experiments.

## CRediT Author Statement

**Irene Setiawan:** Conceptualization, Methodology, Investigation, Formal analysis, Visualization, Project administration, Writing – original draft; **Leanne K. Morgan:** Conceptualization, Supervision, Writing – review & editing; **Crile Doscher:** Supervision, Writing – review & editing; **Kelvin Ng:** Investigation; **Amandine Bosserelle:** Methodology.

## Declaration of Competing Interest

The authors declare that they have no known competing financial interests or personal relationships that could have appeared to influence the work reported in this paper.

## Data Availability

Christchurch shallow groundwater quality survey (Original data) (Mendeley Data).
